# Optimal use of COVID-19 Ag-RDT screening at border crossings to prevent community transmission: A modeling analysis

**DOI:** 10.1371/journal.pgph.0000086

**Published:** 2022-05-16

**Authors:** Joshua M. Chevalier, Karla Therese L. Sy, Sarah J. Girdwood, Shaukat Khan, Heidi Albert, Amy Toporowski, Emma Hannay, Sergio Carmona, Brooke E. Nichols

**Affiliations:** 1 Department of Global Health, Boston University School of Public Health, Boston, Massachusetts, United States of America; 2 Department of Epidemiology, Boston University School of Public Health, Boston, Massachusetts, United States of America; 3 Health Economics and Epidemiology Research Office, Department of Internal Medicine, School of Clinical Medicine, Faculty of Health Sciences, University of the Witwatersrand, Johannesburg, South Africa; 4 Department of Medical Microbiology, Amsterdam University Medical Center, Amsterdam, The Netherlands; 5 Clinton Health Access Initiative, Boston, Massachusetts, United States of America; 6 Foundation for Innovative New Diagnostics, Cape Town, South Africa; 7 Foundation for Innovative New Diagnostics, Geneva, Switzerland; University of Washington, UNITED STATES

## Abstract

Countries around the world have implemented restrictions on mobility, especially cross-border travel to reduce or prevent SARS-CoV-2 community transmission. Rapid antigen testing (Ag-RDT), with on-site administration and rapid turnaround time may provide a valuable screening measure to ease cross-border travel while minimizing risk of local transmission. To maximize impact, we developed an optimal Ag-RDT screening algorithm for cross-border entry. Using a previously developed mathematical model, we determined the daily number of imported COVID-19 cases that would generate no more than a relative 1% increase in cases over one month for different effective reproductive numbers (Rt) and COVID-19 prevalence within the recipient country. We then developed an algorithm—for differing levels of Rt, arrivals per day, mode of travel, and SARS-CoV-2 prevalence amongst travelers—to determine the minimum proportion of people that would need Ag-RDT testing at border crossings to ensure no greater than the relative 1% community spread increase. When daily international arrivals and/or COVID-19 prevalence amongst arrivals increases, the proportion of arrivals required to test using Ag-RDT increases. At very high numbers of international arrivals/COVID-19 prevalence, Ag-RDT testing is not sufficient to prevent increased community spread, especially when recipient country prevalence and Rt are low. In these cases, Ag-RDT screening would need to be supplemented with other measures to prevent an increase in community transmission. An efficient Ag-RDT algorithm for SARS-CoV-2 testing depends strongly on the epidemic status within the recipient country, volume of travel, proportion of land and air arrivals, test sensitivity, and COVID-19 prevalence among travelers.

## Introduction

Severe acute respiratory syndrome coronavirus-2 (SARS-CoV-2), the causative agent of COVID-19, first emerged in Wuhan, China in late 2019. Aided by globalization, the novel coronavirus quickly spread to countries around the world. On the 11th of March 2020, COVID-19 was officially declared a global pandemic by the World Health Organization (WHO). In response to the outbreak, many countries implemented control measures on mobility, such as travel restrictions to and from affected countries, border closures, or quarantine-on-arrival [1].

A key strategy to combat the COVID-19 pandemic is widespread accessible diagnostic testing [2–4]. Countries around the world have implemented travel policies that require international travelers to provide proof of a negative COVID-19 reverse transcription polymerase chain reaction (RT-PCR) test within 48–72 hours of arrival. While this is possible to implement in many high-income countries and for air travel, this is frequently not possible at land-border crossings, particularly in low- and middle-income countries (LMIC), due to frequent cross-border travel and limited access to timely RT-PCR testing. The rate of COVID-19 diagnostic testing in LMICs remains up to 50 times lower than in HICs, while test positivity is far higher, underestimating the true burden of COVID-19 in these regions [5]. Global partnerships have sought to boost testing capacity in African countries specifically, by providing high-quality rapid antigen diagnostic tests (Ag-RDTs) approved for emergency use by the WHO. In September 2020, the Access to COVID-19 Tools (ACT) Accelerator announced that 120 million Ag-RDTs would be made available for LMICs [6]. Ag-RDTs are less costly than RT-PCR tests, do not require laboratory-based infrastructure, can be performed on-site by appropriately trained non-laboratory staff, and provide results within minutes, enabling decentralization of diagnostic testing [4].

In late 2020, South Africa became one of the first countries to deploy Ag-RDTs at points of entry, with little global policy on how to optimize their use and allocation [7, 8]. To better inform the use of Ag-RDTs in this capacity we aimed to develop a generalizable parsimonious algorithm for the use of Ag-RDTs at border crossings to determine the proportion of travelers that would need to be tested with Ag-RDTs to ensure international travel does not increase the baseline community transmission within a 1% margin of error. While such a strategy may provide greater benefit in settings with high volumes of land border crossings or limited RT-PCR testing capacity, the results of this analysis have broad implications for the use of Ag-RDTs in COVID-19 surveillance world-wide.

## Methods

We developed a generalizable Ag-RDT screening strategy for use at border crossings with the goal of preventing an increase in community transmission (relative change in incidence) over a one-month period within the recipient country that is attributable to imported COVID-19 cases, within a 1% margin of error compared to a baseline of zero imports. This algorithm was developed for differing effective reproductive numbers at time *t* (Rt) and COVID-19 prevalence within the recipient country (South Africa in this analysis), under varying levels of Ag-RDT sensitivity, number of daily travelers and COVID-19 prevalence amongst travelers. While most of our analysis maintains recipient country prevalence at 0.1%, corresponding to incidence between epidemic waves when inter-country travel is most likely, we further demonstrate the impact a change in recipient country prevalence has on the maximum allowable daily COVID-19 case importations. The integrated border crossing algorithm determines the proportion of border crossings that would need to be tested per day with Ag-RDTs to prevent the relative 1% increase in transmission.

### Maximum allowable daily COVID-19 importations

**Fig 1:**
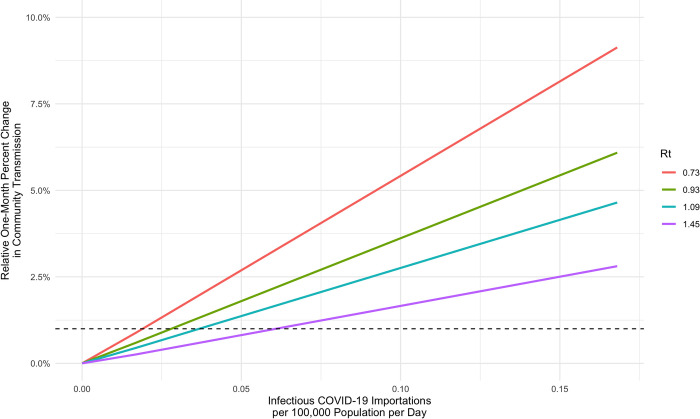
Linear relationship between daily COVID-19 case importations and the relative change in community transmission over the course of one-month by effective reproductive number (Rt) with recipient country COVID-19 prevalence at 0.1%. The horizontal dashed line at 1% represents the threshold of maximum allowable daily COVID-19 importations.

To determine the number of daily infectious COVID-19 case imports that would lead to no more than a relative 1% change in community transmission over a one-month period compared to a baseline of zero imports, we used a COVID-19 susceptible-exposed-infectious-recovered (SEIR) model developed at the Biozentrum, University of Basel [9]. This model has been used previously to make inferences about the spread of COVID-19 in various countries [10]. Their mathematical model allows the user to adjust the number of cases ‘imported’ into the country per day, as well as adjust the level of Rt. To determine the relationship between the number of imported cases per day and community transmission, we exported the relative change in cases over a one-month period for increasing levels of daily SARS-CoV-2 importations (0, 10, 100, 1000 infected persons per day) for four different levels of Rt (Rt = 0.73, 0.93, 1.09, 1.45). This relationship was determined to be perfectly linear. South Africa was the recipient country used in our analysis and country-specific model preset parameters were used (**Table 1**), apart from the effective reproductive number, number of initial cases, imports per day and the infectious period. Other model presets such as number of hospital beds or days spent in ICU that were not relevant to our analysis are further described elsewhere [9]. Within each level of Rt, the number of daily imported cases was linearly related to the total number of cases over a one-month time-period. This linear relationship allowed us to calculate the daily number of COVID-19 importations that would lead to a relative 1% increase in community transmission over the one-month period for all four levels of Rt, as shown in **Fig 1** for a recipient country prevalence of 0.1%.

**Table 1:** SEIR model parameters and inputs.

Parameter	Value(s)
Effective reproductive number	0.73, 0.93, 1.09, 1.45
Recipient country prevalence*	0.1%, 0.2%, 0.5%, 1.0%
Latency period (days)	3
Infectious period (days)	7
Imports per day**	0–1.68
Simulation time range	01 Dec 2020–01 Jan 2021
Number of runs	100

*Recipient country prevalence parameterized by adjusting the initial number of starting cases as a proportion of country population.

**Imports per day per 100,000 population.

### Algorithm parameters

#### Number of international arrivals

The number of international arrivals is expressed as the total number of people entering the country (by land or air) per day per 100,000 population of the host country. The number of international arrivals per 100,000 population of the host country varies substantially depending on the country, stage of the pandemic and levels of international travel.

#### Prevalence of COVID-19

True prevalence of people infectious with SARS-CoV-2 at a given point in time within a country is difficult to measure given the high incidence of asymptomatic infection, underreporting, and limited diagnostic testing capacity in low resource settings. Therefore, we simplified this parameter in the algorithm by varying COVID-19 prevalence (0.1%, 0.2%, 0.5%, 1%) among imports to model how a change in incidence would influence the Ag-RDT screening strategy.

#### Ag-RDT sensitivity

Standard Ag-RDT sensitivity was assumed to be 71% in the base case, as reported in a meta-analysis of 133 clinical and analytical studies evaluating the accuracy of Ag-RDTs [11]. The upper bound of Ag-RDT sensitivity was set at 80%, representing a best-case scenario, based on independent evaluations conducted by the Foundation for Innovative New Diagnostics (FIND) on two WHO approved Ag-RDT tests being made available to LMICs [12, 13]. To address the importance of the SARS-CoV-2 infectiousness profile and viral load trajectory on Ag-RDT performance, the lower bound of test sensitivity set to 50% [11, 14].

#### RT-PCR testing

Airline travelers are often required to present proof of a negative COVID-19 RT-PCR test within 48–72 hours of travel depending on the destination country. A certain percentage of infections will be missed due to testing error or insufficient viral load. Based on a simulation model of 100,000 travelers that assessed a 72-hour pre-travel RT-PCR test, 88% of actively infectious travelers were detected, however, this does not include pre-infectious travelers who may still enter a country [15]. Assuming actively infectious travelers represent 78% of all infected travelers with a pre-infectious period of 2 days and an infectious period of 7 days, a 72-hour pre-flight PCR test will detect 69% of all infectious travelers. We have assumed that 100% of passengers traveling by air will have a negative RT-PCR test within 72-hours prior to travel and that 0% of passengers traveling by land will have a negative RT-PCR test within 72-hours prior to travel in this analysis.

### Interpretation

The algorithm compared the number of undetected COVID-19 cases that would enter the country under the given conditions with the tolerable number of COVID-19 imports calculated for that level of Rt within the recipient country and determines the minimum proportion of arrivals that would need to be screened. We evaluated the effect of testing all land entries, all air entries, or a combination of both, assuming all air travelers are entering with proof of a negative COVID-19 RT-PCR test.

## Results

**Fig 2:**
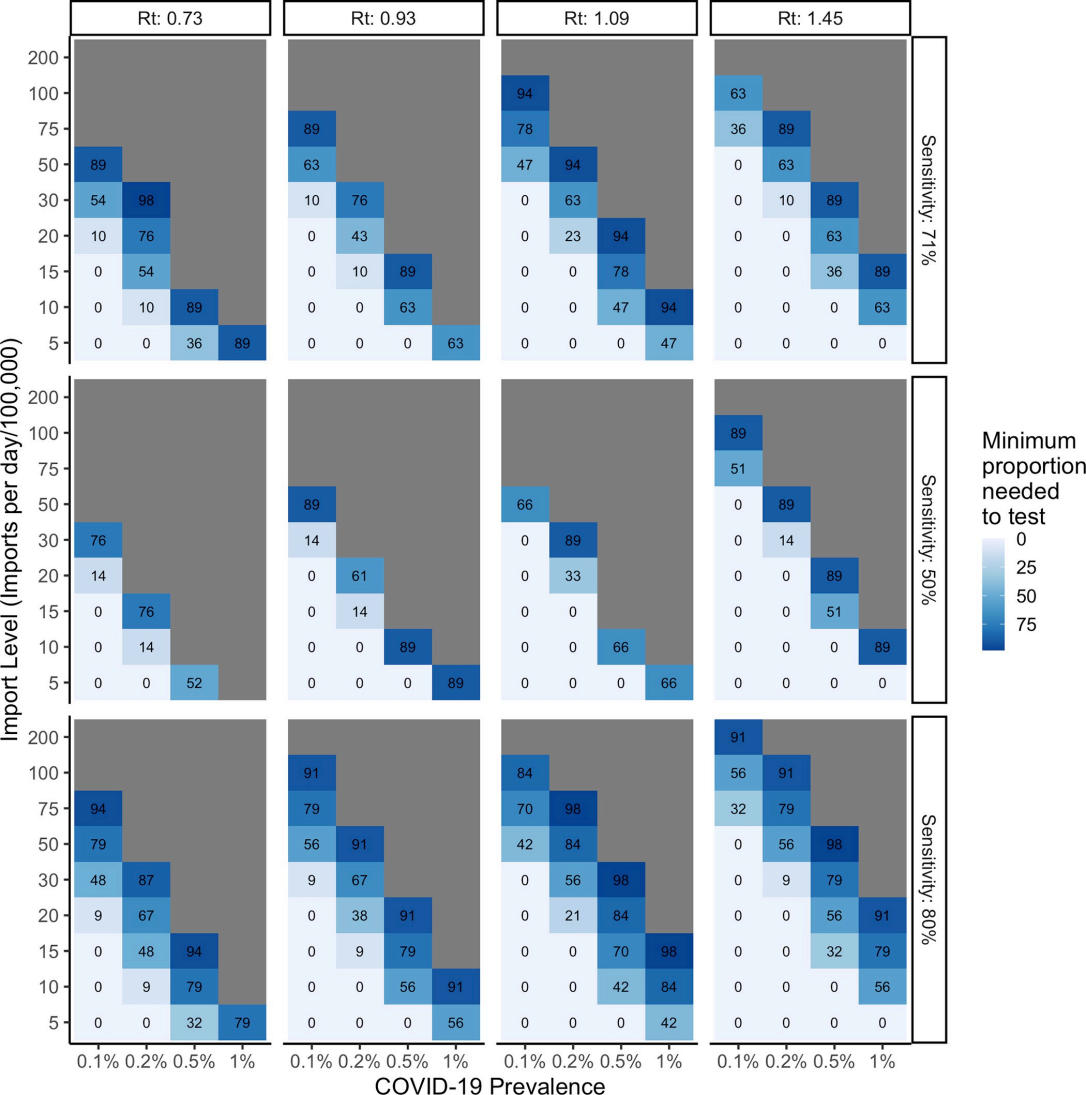
Impact of Ag-RDT test sensitivity on the minimum proportion of all arrivals (land and air) to be screened per day at the border using SARS-CoV-2 Ag-RDT tests. Varied by the reproductive number at time *t*, number of arrivals per day, and prevalence of COVID-19 among arrivals. The number in each cell represents the minimum proportion to be tested, while grey cells indicate Ag-RDT screening under the given conditions (even at 100%) is insufficient to prevent a relative 1% increase in community transmission in the host country over the course of one month.

The minimum proportion of international arrivals required to be screened using Ag-RDTs at the border to prevent a relative 1% increase in community transmission over a one-month period depends on the total number of daily border crossings per 100,000 population of the recipient country, the prevalence of COVID-19 among arrivals, the proportion traveling into the country by land or air, and Ag-RDT sensitivity. As the Rt within the recipient country decreases, the proportion of international arrivals required to be tested increases. Border Ag-RDT screening alone is insufficient at moderate to high volumes of travel, when COVID-19 prevalence among international arrivals is high, and when Ag-RDT sensitivity is low (**Fig 2**). When the Rt is greater than one, Ag-RDT screening of border crossings is unnecessary at lower levels of travel volume and/or lower levels of COVID-19 prevalence among international arrivals. Ag-RDTs with higher sensitivities allow for a wider range of parameters in which a simple Ag-RDT screening strategy would be effective at preventing >1% increase in community transmission over a one-month period in the recipient country.

**Fig 3:**
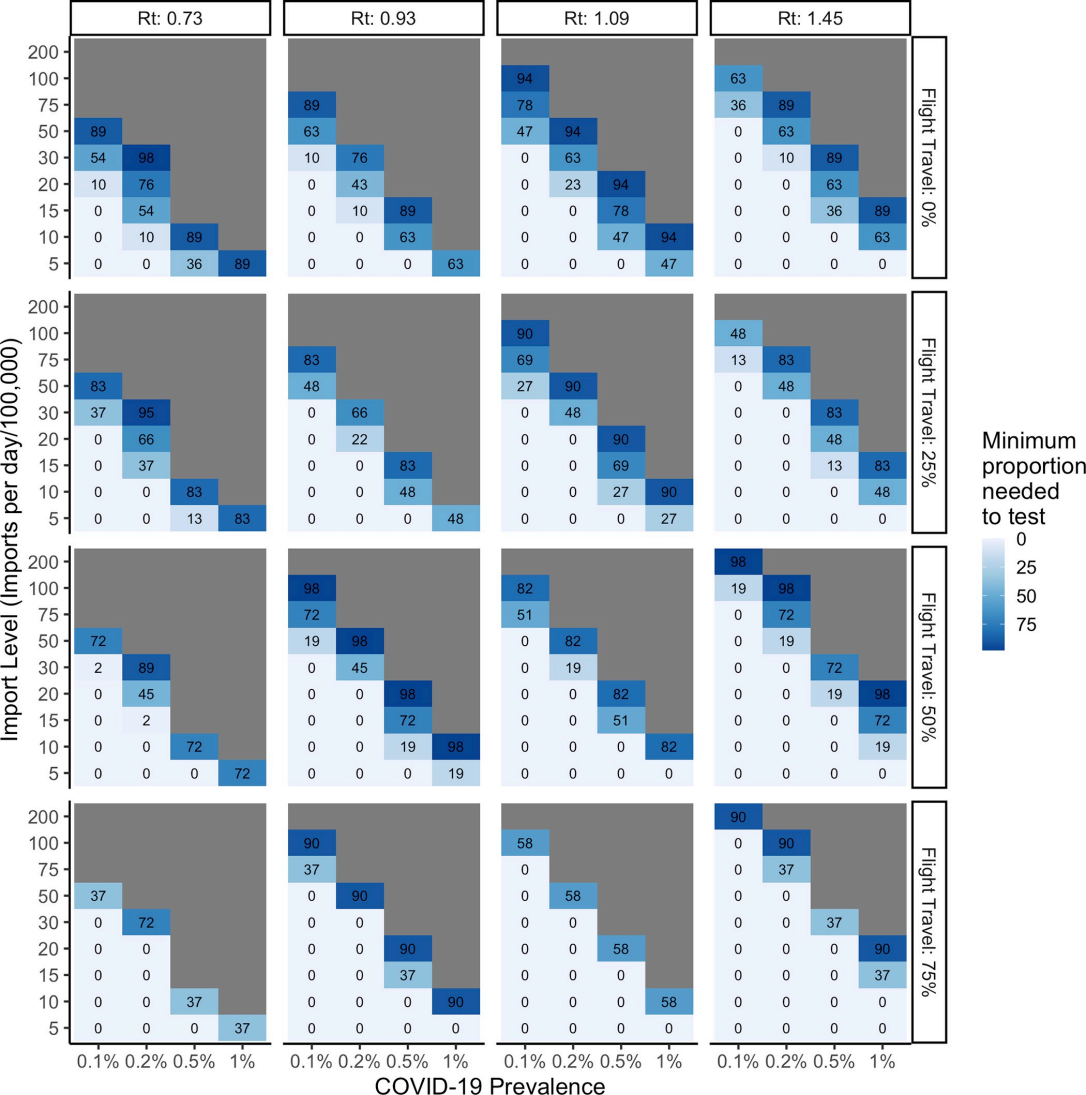
Impact of increasing proportion of flight travel on the minimum proportion of arrivals to be screened per day at land border crossings using SARS-CoV-2 Ag-RDTs when varied by the reproductive number at time *t*, number of arrivals per day, and prevalence of COVID-19 among arrivals. Ag-RDT sensitivity is set to 71%.

**Fig 4:**
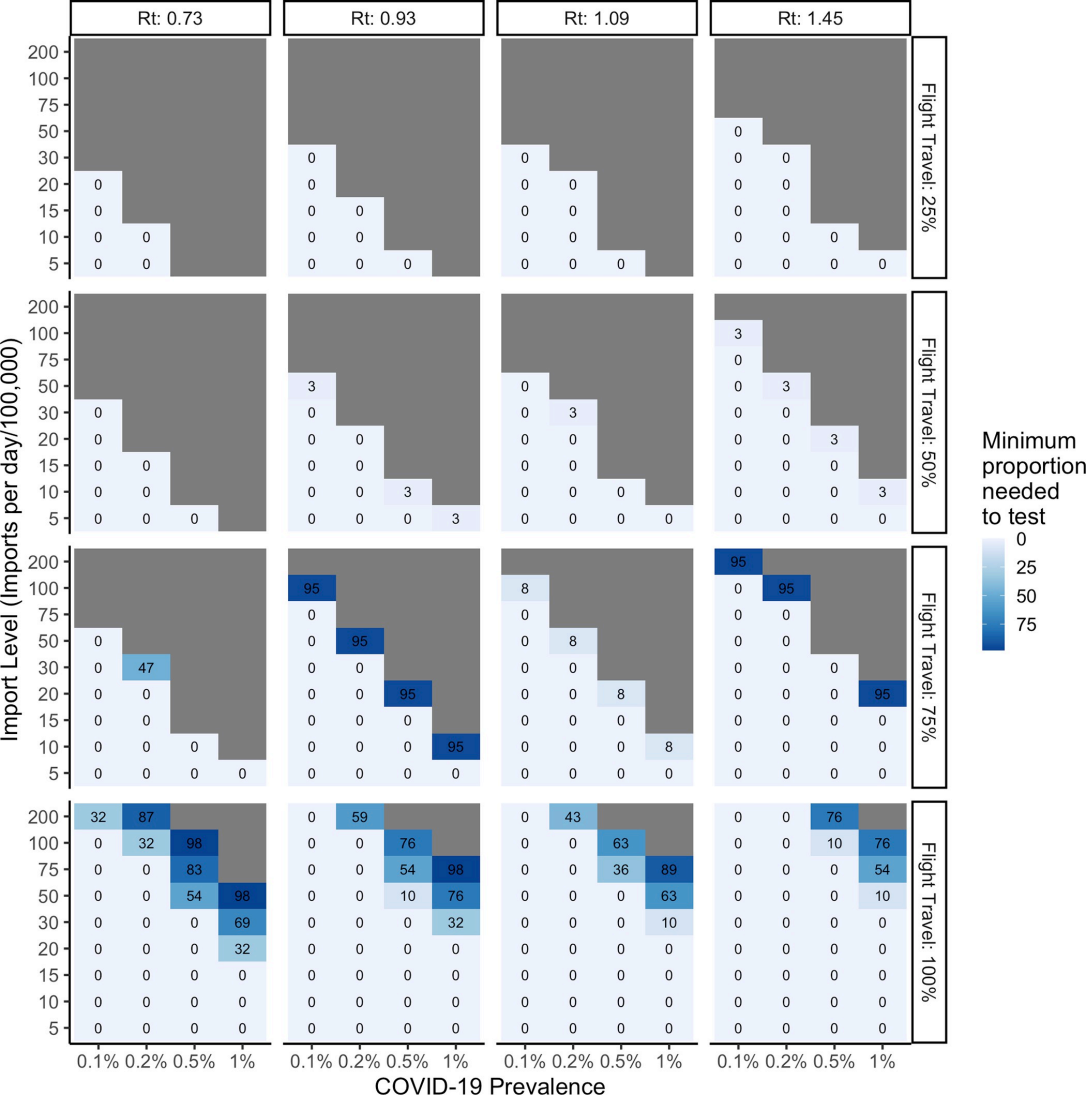
Impact of increasing proportion of flight travel when Ag-RDT screening is only implemented for flight arrivals on the minimum proportion of travelers to be screened with SARS-CoV-2 Ag-RDTs when varied by reproductive number at time t, number of imports per day and SARS-CoV-2 prevalence. Ag-RDT sensitivity is set to 71%.

**Fig 5:**
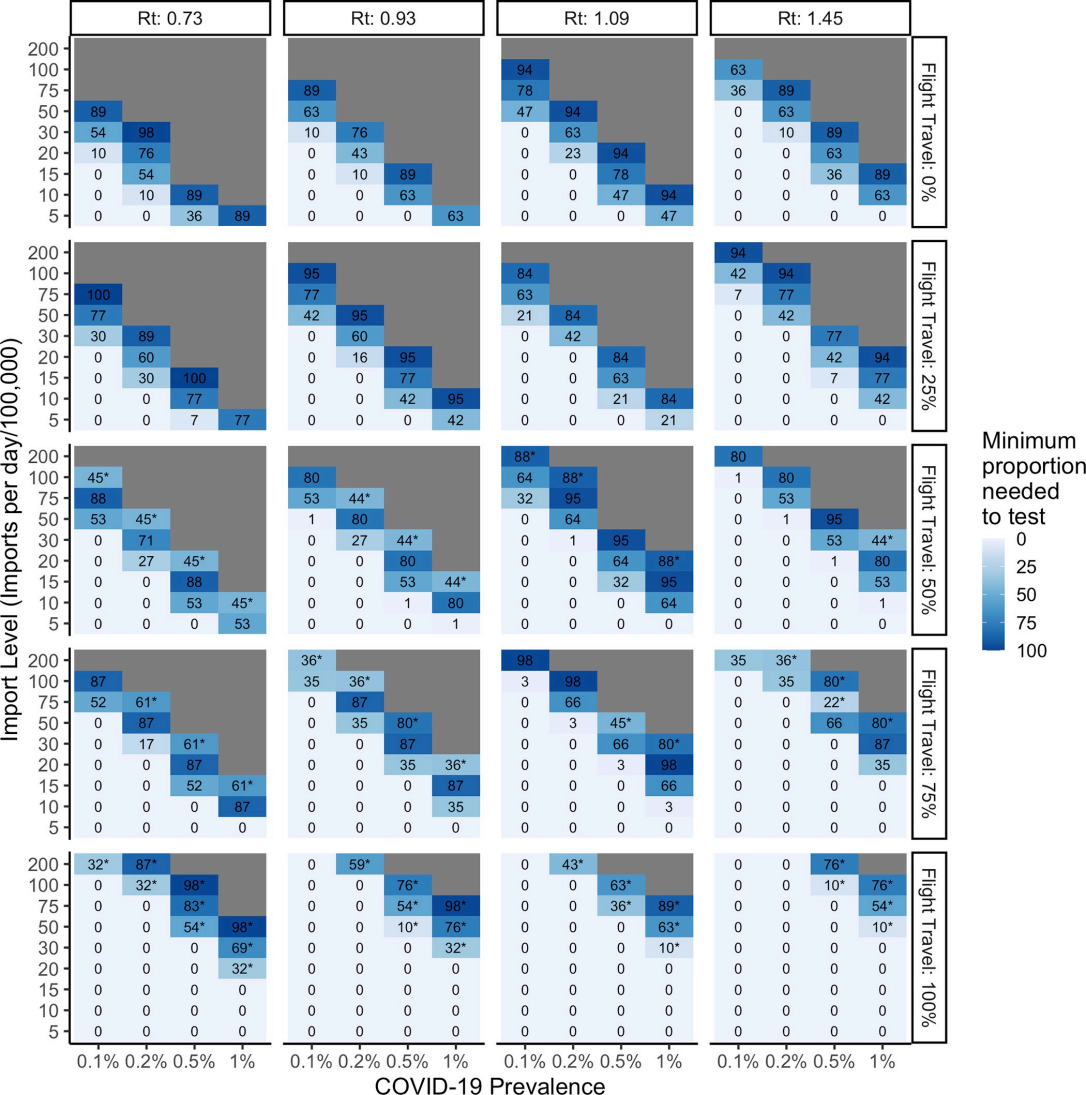
Impact of increasing proportion of flight travel when Ag-RDT screening is first fully saturated at land borders and additionally implemented for flight arrivals. Cells with an asterisk (*) represent the additional proportion of flight arrivals to be screened with SARS-CoV-2 Ag-RDTs following full saturation of land testing. Cells without an asterisk correspond to the minimum proportion of land arrivals required to be tested (Fig 2). Ag-RDT sensitivity is set to 71%.

Importantly, efficiency of the algorithm can be improved by focusing the use of Ag-RDT testing on the borders where arrivals do not have a negative RT-PCR test on arrival (e.g., through many land borders). When the majority of international arrivals into a country are, however, via air, then airports become the only logical place to implement border testing using Ag-RDTs. **Fig 3** illustrates the proportion of land border crossings that need to be screened with Ag-RDTs when screening is only implemented at land crossings, **Fig 4** illustrates the proportion of air travelers that need to be screened with Ag-RDT when only implemented for air arrivals, and **Fig 5** illustrates the proportion of air travelers that would need to be screened in addition to full saturation of land border testing.

**Fig 6:**
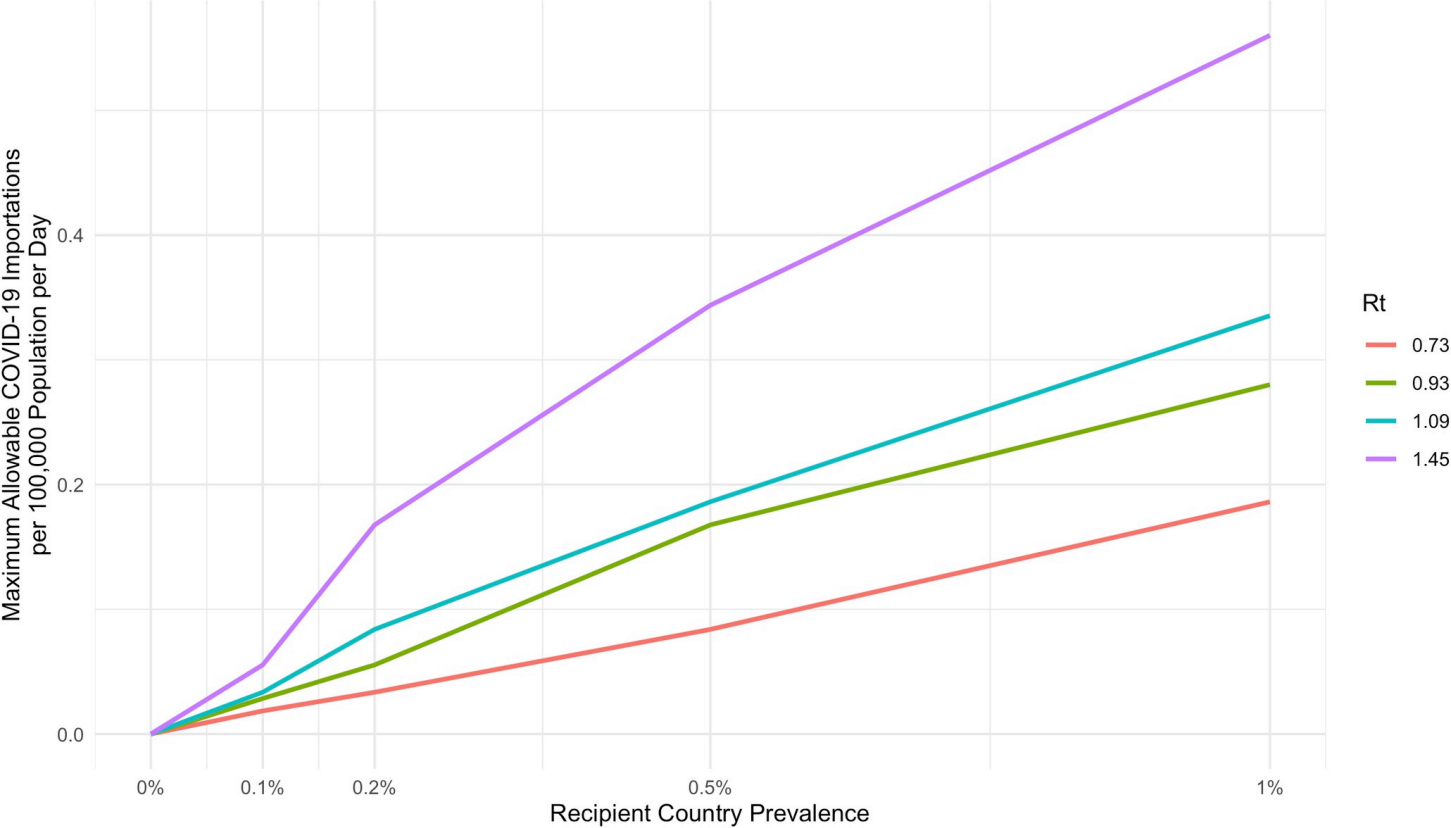
Maximum allowable daily COVID-19 case importations varied by recipient country prevalence and the effective reproductive number at time *t*.

**Figs 2–5** maintain recipient country prevalence at 0.1% as this more closely reflects the case numbers seen between epidemic waves when travel to the country would be most likely. In **Fig 6** we demonstrate the impact of increasing recipient country prevalence on the daily maximum allowable number of COVID-19 importations across all four levels of Rt. As recipient country prevalence increases, the maximum allowable number of daily importations increases, with the magnitude of difference between Rt levels quickly expanding. This would effectively reduce the proportion of arrivals required to be screened with Ag-RDTs at higher levels of recipient country prevalence.

## Discussion

The effectiveness of a SARS-CoV-2 Ag-RDT screening strategy at border crossings depends largely on the current state of the COVID-19 pandemic within the recipient country (represented by Rt and recipient country prevalence), the number of daily international arrivals into the recipient country, the proportion entering by land versus air (given the negative RT-PCR required within 72-hours of air travel), the expected COVID-19 prevalence among arrivals, and the sensitivity of Ag-RDTs. As the Rt and COVID-19 prevalence within the recipient country decrease, the proportion of border crossings required to screen to prevent the relative 1% increase in community transmission increases. As travel volume and COVID-19 prevalence increase, the proportion required to screen increases. At high travel volume and COVID-19 prevalence, the Ag-RDT screening strategy becomes ineffective on its own, especially at lower levels of Rt. In these situations, 100% of arrivals would need to be tested, and then further supplemented by quarantining and additional testing. Border Ag-RDT testing would no longer be required to prevent a relative 1% increase in community transmission when Rt is high and when travel volume and COVID-19 prevalence are low. Recipient country prevalence has a similar effect to Rt, increasing the maximum threshold of daily allowable cases at higher estimates to a point that would no longer require border screening. The Rt and recipient country prevalence together determine how much community transmission is occurring within the country, thus lessening the impact of imported COVID-19 cases. In such situations, Ag-RDTs could be reallocated for other uses and help mitigate internal community transmission.

We demonstrate several scenarios in which the proportion of international arrivals that would require an Ag-RDT test ranges from 0% to 100%. This could be implemented in multiple ways, for example, if Ag-RDT screening were to be implemented for 50% of border crossings, tests could be evenly distributed across points of entry or more heavily allocated to the points of entry where COVID-19 prevalence among travelers is known to be highest, maximizing impact. This could be implemented solely at land borders or a combination of land borders and airports where prior RT-PCR screening was not feasible for travelers. Our results show a narrow range of scenarios where an Ag-RDT screening strategy is sufficient on its own, with increased effectiveness when tests are first saturated at land borders and then further allocated to points of air entry. However, Ag-RDTs have a greater overall impact when implemented for travelers without a prior negative RT-PCR test. The effectiveness of a screening strategy is largely context dependent and could be further enhanced with a combination of additional NPIs in the scenarios where 100% of border screening using Ag-RDTs is insufficient to prevent a relative 1% increase in community transmission over a one-month period.

To the best of our knowledge, this is the first study to examine the role of Ag-RDTs at border crossings, incorporating both land and air imports. A limited number of studies have evaluated the effect of RT-PCR testing on COVID-19 importations [16, 17]. *Russell et al*. modeled the effect of travel restrictions on the importation of COVID-19 cases and their risk to local incidence, finding that travel restrictions have little impact on local epidemics under conditions of high community transmission, but may play an important role when COVID-19 incidence is low within the country [18]. Countries like New Zealand and Australia would not have been able to eliminate community spread without strict border control and containment measures [19, 20]. Modeling of New Zealand’s current arrival protocols with variations concluded that 14-day quarantine on arrival with two consecutive negative RT-PCR tests is the most effective regimen to maintain elimination in the country [20]. These studies suggest testing alone is unlikely to completely eliminate the risk of outbreaks posed from international arrivals. The role of Ag-RDTs in any strategy ultimately depends on the goal of each country. Our analysis is the first attempt at quantifying the amount of testing necessary for a point Ag-RDT screening strategy at border crossings, evaluated against a predetermined tolerable increase in community transmission. Our generalizable algorithm can be used directly by high-, low- and middle-income countries to help inform border testing policy and resource allocation.

Our analysis comes with important assumptions and limitations. First, due to the nature of COVID-19 prevalence, serology studies, and differing testing algorithms by country leading to variations in underreporting, determining the true incidence of COVID-19 at any given instance is not feasible. To address this, we have assumed a maximum incidence of 1% and provide an increasing range up to this point. Second, the algorithm output directly relies on the 1% threshold calculated individually for each level of Rt; however, the 1% margin of error threshold could theoretically be modified to re-define what a tolerable increase in community transmission from outside imports would be for a given country, based on the stage of the epidemic and available resources. Additionally, our model used predetermined thresholds in the context of South Africa but could be calibrated to any country of interest using the same model. However, the overall trends exhibited in our results remain generalizable to scenarios in other countries. Third, the algorithm evaluates the benefit of a single point/once-off Ag-RDT screening strategy on its own and was not analyzed in combination with follow-up testing or other mitigation measures that could enhance its effect, as this falls outside the scope of our analysis. The decentralized nature of Ag-RDT testing, while beneficial, may prove difficult to implement alongside additional strategies, such as contact tracing—particularly in limited resource settings, as tested individuals would not be followed-up with. Fourth, while we attempt to incorporate the infectiousness profile of SARS-CoV-2 by providing a lower bound of Ag-RDT sensitivity at 50%, the nature of the algorithm does not allow for test probabilities based on day of infection. The aim of our analysis was to identify and prevent the importation of infectious travelers but cannot account for those who have an insufficient viral load at the time of testing. Therefore, the 50% measure of test sensitivity can be used as a conservative reference point (**Fig 2**). While we provide a range of estimates in our parameters to capture variation and maintain model sensitivity, if the natural history of SARS-CoV-2 changes, our model may need to be recalibrated, specifically with respect to the SEIR model used to inform the analysis. Finally, if the goal of a given country is to prevent any SARS-CoV-2 importation, Ag-RDT screening is likely to be insufficient and would need to be implemented in combination with quarantine-on-arrival and/or test-to-release measures.

Optimal allocation of Ag-RDTs to border crossings depends strongly on the Rt within the recipient country, volume of travel, proportion of land and air arrivals, test sensitivity, and COVID-19 prevalence among travelers. When travel and incidence are low, implementing a border screening strategy could be beneficial, but when both are high a country may consider reallocating their Ag-RDTs to other testing programs within their country. As with many decisions in this pandemic, the optimal border testing algorithm for a given country will depend on the goal of the testing strategy.
